# RLEP LAMP for the laboratory confirmation of leprosy: towards a point-of-care test

**DOI:** 10.1186/s12879-021-06882-2

**Published:** 2021-11-25

**Authors:** Malkin Saar, Marcus Beissner, Fatih Gültekin, Issaka Maman, Karl-Heinz Herbinger, Gisela Bretzel

**Affiliations:** 1 Division of Infectious Diseases and Tropical Medicine, University Hospital, Ludwig-Maximilians-University (LMU) Munich, Leopoldstrasse 5, 80802 Munich, Germany; 2 Ministère de la Santé, Institut National d’Hygiène (INH), Lomé, Togo

**Keywords:** *Mycobacterium leprae*, Leprosy, NAAT, LAMP, RLEP, Dry-reagent-based, Point-of-care, Household contact

## Abstract

**Background:**

Nucleic acid-based amplification tests (NAAT), above all (q)PCR, have been applied for the detection of *Mycobacterium leprae* in leprosy cases and household contacts with subclinical infection. However, their application in the field poses a range of technical challenges. Loop-mediated isothermal amplification (LAMP), as a promising point-of-care NAAT does not require sophisticated laboratory equipment, is easy to perform, and is applicable for decentralized diagnosis at the primary health care level. Among a range of gene targets, the *M. leprae* specific repetitive element RLEP is regarded as highly sensitive and specific for diagnostic applications.

**Methods:**

Our group developed and validated a dry-reagent-based (DRB) RLEP LAMP, provided product specifications for customization of a ready-to-use kit (intended for commercial production) and compared it against the in-house prototype. The assays were optimized for application on a Genie^®^ III portable fluorometer. For technical validation, 40 “must not detect RLEP” samples derived from RLEP qPCR negative exposed and non-exposed individuals, as well as from patients with other conditions and a set of closely related mycobacterial cultures, were tested together with 25 “must detect RLEP” samples derived from qPCR confirmed leprosy patients. For clinical validation, 150 RLEP qPCR tested samples were analyzed, consisting of the following categories: high-positive samples of multibacillary (MB) leprosy patients (> 10.000 bacilli/extract), medium-positive samples of MB leprosy patients (1.001–10.000 bacilli/extract), low-positive samples of MB leprosy patients (1–1.000 bacilli/extract), endemic controls and healthy non-exposed controls; each n = 30.

**Results:**

Technical validation: both LAMP formats had a limit of detection of 1.000 RLEP copies, i.e. 43–27 bacilli, a sensitivity of 92% (in-house protocol)/100% (ready-to-use protocol) and a specificity of 100%. Reagents were stable for at least 1 year at 22 °C. Clinical validation: Both formats showed a negativity rate of 100% and a positivity rate of 100% for high-positive samples and 93–100% for medium positive samples, together with a positive predictive value of 100% and semi-quantitative results. The positivity rate for low-positive samples was 77% (in-house protocol)/43% (ready-to-use protocol) and differed significantly between both formats.

**Conclusions:**

The ready-to-use RLEP DRB LAMP assay constitutes an ASSURED test ready for field-based evaluation trials aiming for routine diagnosis of leprosy at the primary health care level.

**Supplementary Information:**

The online version contains supplementary material available at 10.1186/s12879-021-06882-2.

## Background

Leprosy caused by *Mycobacterium leprae* is a neglected, chronic infectious disease predominantly affecting the skin and peripheral nerves which is transmitted through the aerial route [1, 2].

The disease is spectral and categorized according to the Ridley–Jopling classification based on the type of lesions and the bacterial load into indeterminate, tuberculoid, lepromatous and three borderline forms. Alternatively, a simplified, field-based classification introduced by the WHO distinguishes paucibacillary (PB, up to five lesions and/or only one nerve trunk involved) and multibacillary (MB, more than five lesions and/or more than one nerve trunk involved) forms. The clinical diagnosis of leprosy can be challenging and the simplified, field-based WHO classification is widely used without applying bacteriological analysis [3]. In the absence of bacteriological evidence misclassification and misdiagnosis leading to inappropriate treatment can occur [4, 5].

To bring back the laboratory diagnostic component into routine practice [5], beyond acid-fast bacilli (AFB) microscopy with its limitations regarding expertise, sensitivity, and specificity [5–7], a range of PCR-based molecular diagnostic tests targeting various gene markers have been applied to the diagnosis of leprosy from skin biopsies and slit skin smears with a positivity rate approaching 50% in PB and 80% in MB cases [5].

Real-time qPCR is considered at least 20 times more sensitive than microscopy [8] and among a range of possible gene targets, the *M. leprae* specific repetitive element RLEP is regarded as highly sensitive, 100% specific and therefore a suitable target for diagnostic applications [5, 6, 9–12].

RLEP belongs to a family of dispersed repeats with unknown function in the genome of *M. leprae*, namely: LEPRPT, LEPREP, REPLEP and RLEP (in ascending order of copy numbers) [13, 14]. Four slightly different RLEP sequences were published: RLEP 1, RLEP 2, RLEP 3 and RLEP 4 (GenBank [pubmed, NCBI], accession numbers FM211192.1, X17151.1, X17153.1 and X17152.1), with RLEP 1 showing most nucleotide differences among these sequences. An *M. leprae* specific central portion (545 bp [15] to 488 bp [13]) is common to all copies of RLEP, and flanked by variable additional sequences (44–100 bp [15] to 113–587 bp [13]). Published copy numbers vary between 28 copies (cp) [15] and 37 cp [13], according to mutations in the primer binding sites. For the genome of Togolese strains as used in this study, the copy number was averaged to 30 cp, determined through two different qPCRs detecting RLEP and 16S rRNA using logarithmic dilutions of plasmid standards as published by Beissner et al. [16]. For one patient with Pakistani origin but living for decades in Munich, Germany, diagnosed and treated at DITM (Division of Infectious Diseases and Tropical Medicine, Munich), the amplifiable RLEP copy number was 23 cp [17].

Our group developed an RLEP qPCR on nasal swabs constituting a noninvasive sampling technique facilitating early diagnosis of leprosy cases as well as laboratory assessment of contact persons [16]. Nasal swabs have been effectively applied for PCR-based diagnosis and are commonly used to investigate nasal carriage leading to subclinical infection of *M. leprae* in household contacts (HHC) [12, 18–26].

Although recognizing the superiority of PCR compared to other diagnostic techniques, previous WHO recommendations did not include PCR for laboratory confirmation of cases or screening of contacts in the field as PCR assays would be difficult to perform in field settings, lack standardization, are not commercially available and require technical and laboratory expertise. Recent policy developments however, such as the scheduled implementation of laboratory diagnostics for the period of 2026–2030 in the upcoming WHO Global Leprosy Strategy 2021–2030, the appraisal of the necessity for effective and affordable diagnostic tests as expressed by the Technical Advisory Group (TAG) and its support for improvement of laboratory capacity including molecular diagnostics, as well as the recent assessment of research priorities by the Global Partnership for Zero Leprosy giving top priority to the development of diagnostic tests constitute a new phase for the role of molecular biology for the diagnosis of leprosy [1, 27–29].

Loop-mediated isothermal amplification (LAMP), a promising nucleic acid-based (NAAT) point-of-care (POC) technology applicable for decentralized diagnosis at the primary health care level, has the potential to change the situation. To overcome PCR limitations LAMP was developed as a field-friendly and cost-effective diagnostic tool providing advantages such as no requirements for sophisticated laboratory equipment and skills, the potential for simplified DNA extraction methods, naked-eye detection of amplicons, and the use of (freeze-) dried reagents [30].

Several studies have shown LAMP as a useful tool for the rapid detection of bacterial, parasitic and viral pathogenic agents of infectious diseases including assays for 16 neglected tropical diseases such as leishmaniasis, schistosomiasis, Buruli ulcer, helminthic diseases or yaws [31–39]. Besides a range of in-house assays, commercial LAMP diagnostic kits developed by Eiken (Eiken Chemical Co., Tokyo, Japan) for human African trypanosomiasis (HAT) [40], tuberculosis (TB) [41], malaria [42–44], and leishmaniasis [45, 46], as well as the centrifugation-free Illumigene assay for malaria genus-level detection developed by Meridian Biosciences (Cincinnati, USA) are available [47, 48].

The LAMP technique employs a *Bacillus stearothermophilus* (*Bst*) derived DNA polymerase and a set of four to six primers (two inner and two outer primers at minimum, and to speed up the reaction and augment sensitivity, if designable, a pair of loop-primers) that recognize six distinct sequences of the target DNA, which makes them highly specific to the target. The LAMP primers can be designed via the user-friendly online platform Primer Explorer V4 software [49] by Eiken. LAMP amplifies a few copies of DNA to 10^9^ cp in less than 30 min with high efficiency [30]. The mechanism behind the LAMP reaction involves three major steps: initiation, cycling amplification, and elongation. Typically, the reaction begins with the initial step, i.e. the binding of the inner primers, followed by strand displacement DNA synthesis by the outer primers. Subsequently, the cyclical amplification step and elongation occur [30]. The auto strand displacement properties of the *Bst* polymerase enable using a heating block or a normal water bath maintained at a constant temperature for the amplification reaction instead of thermal cycling. The appearance of e.g. magnesium pyrophosphate precipitate is indicative for amplification of the target DNA, but amplicon binding fluorophores are also available. Real-time turbidimetry facilitates the quantification of the template DNA in the reaction and allows the analysis of minute quantities of DNA. Furthermore, LAMP amplicons can be analyzed using agarose gel electrophoresis and/or simple colorimetric naked-eye visualization closed detection systems or real-time fluorometry. As a result of these properties, the major advantage of LAMP is its application in the field or other resource-limited settings [30, 34, 50].

Four LAMP assays were recently designed for detecting *M. leprae*: Garg et al., Jiang et al. and Joshi et al. each designed RLEP or 16S LAMP assays, respectively, that required only standard consumables and a constant temperature water bath or heating block. Naked-eye detection was achieved via DNA intercalating dyes (colorimetric detection)—in case of Joshi et al. with a closed tube technology to minimize the risk of cross-contamination—and additional visual detection of turbidity and bridge flocculation assay as used by Garg et al. For further confirmation also agarose gel electrophoresis was used by Garg et al. and Jiang et al. [51–53]. Joshi et al. additionally designed a RLEP multiplex LAMP (m-LAMP) for differential detection of *M. leprae* and *Leishmania donovani* using the portable real-time Genie^®^ II fluorometer from OptiGene (Horsham, United Kingdom) for amplification and detection of the LAMP products [54].

To the best of our knowledge no LAMP detecting *M. leprae* was described that uses dried reagents not requiring a continuous cold or freezing chain. Based on own experience with the development of a dry-reagent-based (DRB) IS*2404* LAMP for decentralized diagnosis of Buruli ulcer in cooperation with the Foundation for Innovative Diagnostics (FIND) which currently awaits clinical validation in Togo [55], our group designed and validated the first RLEP DRB LAMP for the diagnosis of leprosy at point-of-care, provided product specifications for customization of a ready-to-use kit and compared it against the in-house prototype.

## Methods

This study aimed to establish, optimize and validate a leprosy-specific RLEP-detection-based LAMP assay employing lyophilized reagents (i.e. in-house RLEP DRB LAMP) transformable into a commercially produced ready-to-use POC kit (i.e. ready-to-use RLEP DRB LAMP).

### Ethical approval

The study involves human tissue from Togolese, Ghanaian and German patients.

For Togolese and Ghanaian patients, samples were collected in the course of two studies, approved by the national Togolese (Université de Lomé, and Ministry of Health, 012/2012/CBRS) and the Ghanaian KNUST (Kwame Nkrumah University of Science and Technology; NMIMR-IRB CPN 116/12-13) Ethics Committees. Written informed consent (IC) was obtained from all Togolese and Ghanaian study participants and/or their legal representatives if below 18 years of age.

All German patients sought medical advice at our outpatient department and signed a consent form approving the collection of data and samples before they were subjected to routine clinical and laboratory procedures. The form has been approved by the institutional ethics review board of the Medical Centre of the University of Munich (LMU).

### LAMP—primer set

A set of RLEP LAMP primers was pre-designed with PrimerExplorer V4 from Eiken Chemical, Tokyo, Japan [49]. On this basis and analyzing DNA sequences of the *M. leprae* specific repetitive element (RLEP, GenBank, NCBI) LAMP primers for the detection of RLEP sequences from *M. leprae* derived from different clinical samples of various geographic origins were manually optimized for a customized application. The primer set consisted of a pair of inner and outer primers named RLEP FIP, RLEP BIP and RLEP F3, RLEP B3 specific for the RLEP sequence in the *M. leprae* genome. The specificity of the primers was confirmed with the basic local alignment search tool (BLAST, GenBank, NCBI) [56].

RLEP F3 has a forward complementary sequence, RLEP B3 has a reverse complementary sequence and RLEP FIP, as well as RLEP BIP, show a more complex sequence construction binding each at two different positions which results in loop structures typical for LAMP.

**Fig. 1:**
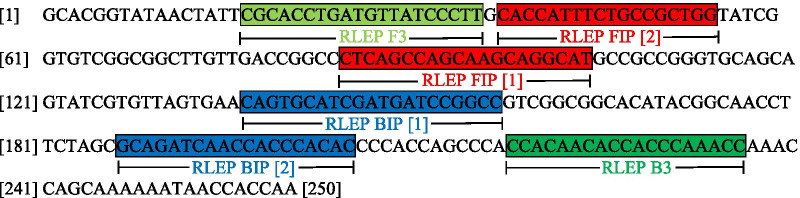
Primer set and location in the RLEP sequence of *M. leprae* Br4923. Primers RLEP FIP and RLEP BIP contain two sequences each binding at different positions ([position 1] and [position 2])

Primer positions within the RLEP sequence of the leprosy genome (*Mycobacterium leprae* Br4923, GenBank Accession No.: FM211192.1) are displayed in Fig. 1.

Primer sequences:

RLEP F3: 5ʹCGCACCTGATGTTATCCCTTʹ3

RLEP FIP: 5ʹATGCCTGCTTGCTGGCTGAG [1] CACCATTTCTGCCGCTGG [2]ʹ3

RLEP BIP: 5ʹCAGTGCATCGATGATCCGGCC [1] GTGTGGGTGGTTGATCTGC [2]ʹ3

RLEP B3: 5ʹGGTTTGGGTGGTGTTGTGGʹ3

### LAMP—optimizing the protocol

After primer design, a protocol for the newly designed LAMP was established and optimized on a qPCR device (CFX 96; BioRad Laboratories, Hercules, USA) using duplicate measurements of RLEP standard 10^7^ (GenExpress, Berlin, Germany; described below in the samples section).

As a dry-reagent-based NAAT depends crucially on a freeze-dryable master mix, based on our experience from previous projects we compared two master mixes, namely Isothermal Mastermix ISO-DR-001 and Isothermal Mastermix ISO-DR-004 from OptiGene in a first test. For a second test, as our own experience differed slightly from the manufacturer’s protocol, different ratios of the inner (RLEP FIP/BIP [20 µM]) and outer primers (RLEP F3/B3 [5 µM]) were compared (inner primers: outer primers 1:1 against 2:1). For a third test, eight different annealing temperatures were compared using a temperature gradient: 68.6 °C, 68.1 °C, 67.0 °C, 65.0 °C, 62.6 °C, 60.6 °C, 59.3 °C and 58.6 °C. For all three tests each the mean, standard deviation (SD) and coefficient of variation (CV) were used for comparison. For the resulting protocol please refer to Additional file 1—in-house “wet” RLEP LAMP run protocol.

### DRB LAMP—freeze-drying

The optimized LAMP protocol was freeze-dried in our department (in-house lyophilization protocol). Then, based on our experience with the development of a DRB IS*2404* LAMP for the diagnosis of Buruli ulcer, according to our specifications the same LAMP protocol was lyophilized by Amplex (Amplexdiagnostics GmbH, Gars am Inn, Germany; ready-to-use lyophilization protocol), to generate simple ready-to-use LAMP test strips for comparison with the in-house lyophilization protocol and eventually future application at point-of-care [55].

#### DRB LAMP—reaction mix for lyophilization

One DRB LAMP reaction mix contained 54 µl primer mix (i.e. 18 µl RLEP FIP [20 µM], 18 µl RLEP BIP [20 µM], 9 µl RLEP F3 [5 µM] and 9 µl RLEP B3 [5 µM]; TibMolBiol, Berlin, Germany) and 90 µl master mix (Isothermal Mastermix ISO-DR-004 [OptiGene] re-suspended with PCR grade water).

#### In-house lyophilization protocol

The reaction mix for the in-house RLEP DRB LAMP was lyophilized in-house into 0.5 ml screw cap microtubes (SARSTEDT, Nümbrecht, Germany) with the freeze dryer Alpha 1–2 LDplus (Christ Martin, Osterode, Germany) for 20 h at 0.31 mbar. For the whole protocol please refer to Additional file 2—in-house lyophilization protocol. All reaction mixes for the in-house RLEP DRB LAMP were stored at 4 °C in the dark.

#### Ready-to-use lyophilization protocol

The reaction mix for the ready-to-use RLEP DRB LAMP was commercially lyophilized by Amplex directly in the wells of the LAMP strips (8-well Genie^®^ Strips, OptiGene). The test is currently for research use only; CE certification can be sought upon demand.

### DRB LAMP—run preparation and protocol

For the in-house as well as the ready-to-use lyophilization protocol the reaction mixes for the in-house as well as the ready-to-use RLEP DRB LAMP were resolved as described below, and the samples and controls were added to run the test on a Genie^®^ III fluorometer (OptiGene), which is a hand-held, battery-driven device for isothermal amplification and real-time fluorescence detection at the point-of-care [57]. Please also refer to the Additional file 3—in-house RLEP DRB LAMP run protocol and Additional file 4—ready-to-use RLEP DRB LAMP run protocol.

#### In-house RLEP DRB LAMP—run preparation and materials

For testing of samples with the in-house RLEP DRB LAMP, 135 µl buffer ISO-DR-004 (OptiGene) and 72 µl PCR grade water were added to the lyophilized in-house RLEP DRB LAMP reaction mix to dissolve it to the total amount of 207 µl. In each well of an 8-well Genie^®^ Strip, 23 µl dissolved reaction mix was pipetted and 2 µl template was added to a final reaction volume of 25 µl.

#### Ready-to-use RLEP DRB LAMP—run preparation and materials

For testing of samples with the ready-to-use RLEP DRB LAMP 12.5 µl buffer ISO-DR-004 and 7.5 µl water were pipetted directly in the wells of the 8-well Genie^®^ Strip to the ready-to-use RLEP DRB LAMP reaction mix. Then, 5 µl template was added to reach a final reaction volume of 25 µl.

#### DRB LAMP—run protocol

The Genie^®^ III fluorometer provides space for one 8-well Genie^®^ Strip, so up to six samples can be processed together with 1 negative and 1 positive control.

**Fig. 2:**
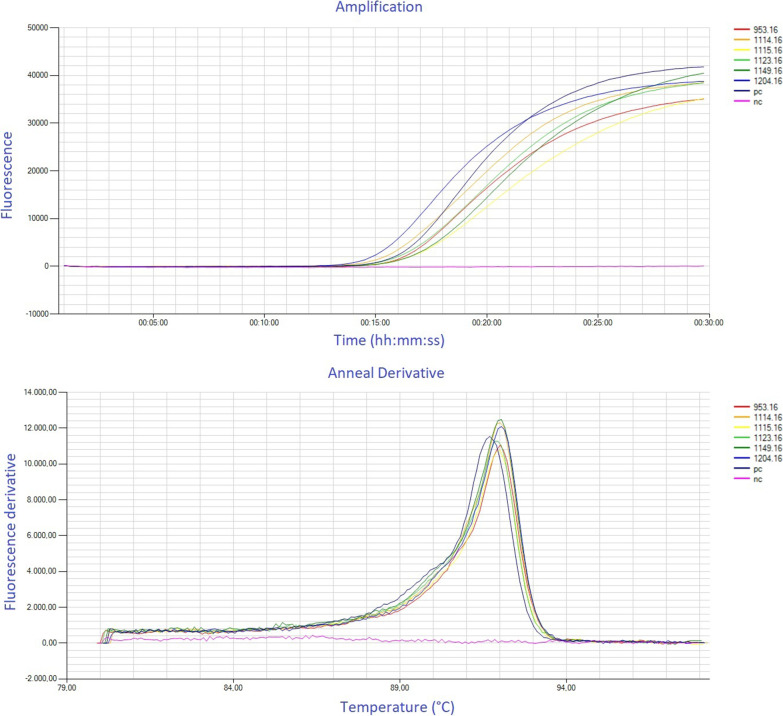
Example of a Genie^®^ III run including the corresponding modified melting curve analysis. 953.16–1204.16 = samples (tested positive); PC = positive control; NC = negative control

Overall, one test had a turn-around time of 45 min, consisting of 10 min hands-on for preparation and 35 min for the run protocol on the Genie^®^ III fluorometer (30 min at 65 °C for amplification and detection, then heating to 98 °C for 1 min and reducing the temperature in steps of 0.05 °C/s to 80 °C for modified melting curve analysis). In Fig. 2 an example of a run is provided.

### Storage testing

The shelf life of the in-house RLEP DRB LAMP was calculated based on accelerated ageing, where the effect of ageing is simulated in a heating cabinet. The calculation of the presumed storage time is based on Arrhenius’ equation, which simply states that a 10 °C increase in temperature doubles the rate of a chemical reaction. To give an example: if a kit is stable for 60 days at 48 °C it is most likely also stable for 1 year at ambient temperature (22 °C). The accelerated ageing calculator applied can be found at iso-inc.com [58].

For testing of accelerated ageing, in-house RLEP DRB LAMP tubes were stored for up to 120 days at 48 °C dry and dark in a heating cabinet (Heraeus BS042E, Thermo Electron Corp. Waltham, USA), to calculate the shelf-life at ambient temperature. After 15, 30, 60 and 120 days in-house RLEP DRB LAMP tubes were tested with a standard series RLEP Std 10^7^–10^3^ (GenExpress, Berlin, Germany; described below in “Samples” section). If the standard samples were tested positive, the experiment was continued; if one or more of the standard samples were tested negative, the accelerated ageing time was defined as exceeded, and the experiment aborted.

Additionally, the shelf life at 4 °C was tested directly. Therefore in-house RLEP DRB LAMP tubes were stored for up to 1 year at 4 °C dry and dark in a laboratory refrigerator. After 3, 6 and 12 months each in-house RLEP DRB LAMP tube was tested as described for the accelerated ageing.

The storage of the ready-to-use RLEP DRB LAMP was assessed by Amplex using an internal protocol.

### Samples

For technical and clinical validation RLEP standards, culture extracts and a wide range of clinical samples from various patients and control groups were tested.

### RLEP plasmid standards

Lyophilized RLEP plasmid standards used for optimization, storage testing and technical validation (RLEP Std 10^8^; GenExpress, Berlin, Germany) were resuspended with DNA hydration solution (Qiagen, Hilden, Germany), according to the manufacturer’s instructions (GenExpress). A tenfold serial dilution (RLEP Std 10^7^–10^1^) was prepared by successive diluting of 10 µl RLEP Std in 90 µl DNA hydration solution. The 10^8^ dilutions were aliquoted and stored at − 20 °C for a maximum of 30 days. Thawed aliquots and tenfold serial dilutions were stored at 4 °C and used within 1 week.

### Clinical samples

A wide range of clinical samples (nasal and buccal swab samples, slit skin smears, biopsies, fine needle aspirates [FNAs], skin scrapings) from non-exposed and endemic controls as well as patients with different diseases were tested.

#### Collection of clinical samples

Clinical samples from untreated MB leprosy patients diagnosed according to the WHO classification and treated in Togo after sample collection, MB leprosy patients diagnosed and treated (prior to sample collection) in Germany, endemic controls from Togo and Germany and healthy non-exposed controls from Germany were collected as previously described [16, 59, 60]. Briefly, nasal and buccal swab samples were collected with custom-made swabs (bio-Budget, Krefeld, Germany), slit skin smears were collected from ear lobes and slit skin smears and biopsies were collected from the edges of lesions. All samples were stored in 700 µl cell lysis solution (CLS, Qiagen) and transported at ambient temperature to the respective laboratory (INH [Institut National d’Hygiène, Lomé] in Togo and DITM in Germany).

Clinical samples from patients with Buruli ulcer disease (BUD) originating from Togo and Ghana were collected as previously described [60] and transported to the respective laboratory (INH in Togo and KCCR [Kumasi Centre for Collaborative Research in Tropical Medicine, Kumasi] in Ghana). Briefly, swabs were collected by circling the entire undermined edges of ulcerative lesions and transported in 700 µl CLS. FNAs were collected from the centre of non-ulcerative lesions or undermined edges of ulcerative lesions including necrotic tissue and transported in 300 µl CLS.

Skin scrapings and biopsies from patients with cutaneous leishmaniasis diagnosed in the outpatient department of DITM were transported in 300 µl CLS to the laboratories of DITM.

#### Extraction of samples

All clinical samples from patients with cutaneous leishmaniasis were extracted using a modified protocol for the “Puregene tissue kit” from Qiagen (Protocol: “DNA Purification from Tissue Using the Gentra Puregene Tissue Kit”, Gentra Puregene Handbook 12/2014). Briefly, the tissue was placed into 300 µl CLS and 1.5 µl Puregene proteinase K (20 mg/ml) was added for incubation at 55 °C overnight. Subsequently, 100 µl Protein Precipitation Solution was added on ice and after a centrifugation step, the supernatant was poured to 300 µl isopropanol. After centrifugation the DNA pellet was washed in 70% ethanol, air dried, and 50 µl DNA hydration solution was added to dissolve the pellet.

All other samples (including culture supernatants in 700 µl CLS) were extracted employing the Gentra Puregene method (Qiagen) as previously described [16].

All extractions included extraction controls for the subsequent (q)PCR as routinely conducted in the accredited laboratories of DITM.

#### Testing of clinical samples

All clinical samples from leprosy patients were tested with RLEP qPCR (as initially designed by Truman et al. [14] and optimized by Beissner et al. [16]).

Additionally, direct DNA sequencing of 21 samples as indicated in Tables 1 and 2 was performed to confirm the presence of *M. leprae* DNA in the respective samples and to determine the RLEP copy number of *M. leprae* strains from patients with Togolese and Pakistani origin for exact quantification of the number of bacilli in clinical samples by RLEP qPCR [16].”

**Table 1:** Number, type and origin of samples used for technical validation of the RLEP DRB LAMP (n = 65)

Purpose	No	Obtained from	Sample type (No)	Origin (No)
“Must detect RLEP” samples (n = 25)	25	Clinical samples from Leprosy patients (RLEP qPCR confirmed; seven samples additionally confirmed by direct DNA sequencing)	Nasal swab (14)	INH^a^ (10)
				DITM^b^ (4)
			Slit skin smear (10)	INH^a^ (8)
				DITM^b^ (2)
			Skin biopsy (1)	DITM^b^ (1)
“Must not detect RLEP” samples (n = 40)	12	Closely related mycobacterial species (confirmed by direct DNA sequencing): *Mycobacterium avium; M. chelonae; M. fortuitum; M. gordonae; M. intracellulare; M. kansasii; M. lentiflavum; M. marinum; M. smegmatis; M. szulgai; M. tuberculosis; M. xenopi*	Culture extract (12)	National Reference Centre for Mycobacteria Borstel Germany (12)
	2	Cultures from patients with Buruli ulcer disease (*Mycobacterium ulcerans*)	Culture extract (2)	DITM^b^ (2)
	4	Clinical samples derived from qPCR confirmed patients with Buruli ulcer disease (BUD)	Nasal swab (2)	KCCR^c^ (2)
			Fine needle aspirate (FNA) (2)	KCCR^c^ (1)
				INH^a^ (1)
	4	Clinical samples from patients with PCR confirmed cutaneous leishmaniasis: *Leishmania braziliensis; L. donovani complex; L. mexicana; L. tropica*	Skin biopsy (3)	DITM^b^ (3)
			Skin scraping (1)	DITM^b^ (1)
	12	Endemic controls: clinical samples from suspected leprosy cases, RLEP qPCR negative	Nasal swab (5)	INH^a^ (4)
				DITM^b^ (1)
			Buccal swab (2)	INH^a^ (2)
			Slit skin smear (4)	INH^a^ (4)
			Skin biopsy (1)	DITM^b^ (1)
	6	Healthy, non-exposed controls: Clinical samples from non-exposed individuals (DITM laboratory staff)	Nasal swab (5)	DITM^b^ (5)
			Buccal swab (1)	DITM^b^ (1)

^a^INH = Institut National d’Hygiène, Lomé, Togo

^b^DITM = Division of Infectious Diseases and Tropical Medicine (outpatient department and accredited diagnostic laboratories), Munich, Germany

^c^KCCR = Kumasi Centre for Collaborative Research in Tropical Medicine, Kumasi, Ghana

**Table 2:** Number, type and origin of samples used for clinical validation of the RLEP DRB LAMP (n = 150)

Sample category	No	Sample type (No)	Origin (No)
RLEP qPCR high-positive samples of MB leprosy patients (five samples additionally confirmed by direct DNA sequencing)	30	Nasal swab (24)	Togo, INH^a^ (19)
			Germany, DITM^b^ (5)
		Slit skin smear (6)	Togo, INH^a^ (5)
			Germany, DITM^b^ (1)
RLEP qPCR medium-positive samples of MB leprosy patients (six samples additionally confirmed by direct DNA sequencing)	30	Nasal swab (24)	Togo, INH^a^ (13)
			Germany, DITM^b^ (11)
		Slit skin smear (5)	Togo, INH^a^ (4)
			Germany, DITM^b^ (1)
		Skin biopsy (1)	Germany, DITM^b^ (1)
RLEP qPCR low-positive samples of MB leprosy patients (10 samples additionally confirmed by direct DNA sequencing)	30	Nasal swab (26)	Togo, INH^a^ (12)
			Germany, DITM^b^ (14)
		Buccal swab (2)	Togo, INH^a^ (2)
		Slit skin smear (2)	Togo, INH^a^ (1)
			Germany, DITM^b^ (1)
RLEP qPCR negative samples of endemic controls (suspected leprosy cases)	30	Nasal swab (13)	Togo, INH^a^ (12)
			Germany, DITM^b^ (1)
		Buccal swab (5)	Togo, INH^a^ (5)
		Slit skin smear (11)	Togo, INH^a^ (11)
		Skin biopsy (1)	Germany, DITM^b^ (1)
RLEP qPCR negative samples of healthy non-exposed controls (DITM laboratory staff)	30	Nasal swab (30)	Germany, DITM^b^ (30)

^a^INH = Institut National d’Hygiène, Lomé, Togo

^b^DITM = Division of Infectious Diseases and Tropical Medicine (outpatient department and accredited diagnostic laboratories), Munich, Germany

#### Determination of bacillary load of clinical samples

Routinely, exact quantification of the number of bacilli per extract, as recently described by Beissner et al. was performed [16]. Briefly, a standard curve of the RLEP qPCR was generated using serial dilutions (10^7^–10^3^ copies) of plasmid standards to estimate the starting quantity (SQ) of clinical samples. The bacillary load (BL) was calculated by BL = (SQ × [volume of DNA extract/volume of template])/copy number. The bacillary load of *M. leprae* was grouped into three categories: high-positive (i.e. > 10.000 bacilli/extract), medium-positive (i.e. 1.001–10.000 bacilli/extract) and low positive (i.e. 1–1.000 bacilli/extract).

#### Samples used for technical validation

For technical validation, RLEP plasmid standards as well as 25 “must detect RLEP” samples from Togo (n = 18) and Germany (n = 7), all RLEP qPCR positive and with a bacillary load of at least 3.000 bacilli/extract, were used (Table 1).

Also, 40 “must not detect RLEP” samples were tested. These consisted of a set of culture extracts from closely related mycobacteria (n = 14), clinical samples derived from IS*2404* qPCR confirmed BUD patients from Togo (n = 1) and Ghana (n = 3) (PCR as first described by Fyfe et al. [61] and modified by Beissner et al. [62]), clinical samples derived from patients with PCR confirmed cutaneous leishmaniasis (n = 4; PCR as described by Schönian et al. [63]), as well as from endemic controls, i.e. RLEP qPCR negative suspected leprosy cases from Togo (n = 10) and Ghana (n = 2) and healthy non-exposed controls, i.e. DITM laboratory staff (n = 6) (Table 1).

#### Samples used for clinical validation

For clinical validation, 150 clinical samples were tested: 30 RLEP qPCR high-positive samples of MB leprosy patients from Togo (n = 24) and Germany (n = 6), 30 RLEP qPCR medium-positive samples of MB leprosy patients from Togo (n = 17) and Germany (n = 13), 30 RLEP qPCR low-positive samples of MB leprosy patients from Togo (n = 15) and Germany (n = 15), 30 samples from endemic controls (suspected leprosy cases, RLEP qPCR negative from Togo (n = 28) and Germany (n = 2) and 30 samples from healthy non-exposed controls (non-exposed DITM laboratory staff from Germany, RLEP qPCR negative) (Table 2).

### Technical validation

For the technical validation of both the in-house and the ready-to-use RLEP DRB LAMP formats, the limit of detection, sensitivity, specificity as well as the inter- and intra-assay variations were determined following the MIQE guidelines for qPCR, as no guidelines exist for validation criteria of the LAMP format [64].

#### LOD

The analytical sensitivity was determined as the lower limit of detection (LOD, i.e. lowest concentration with > 95% samples tested positive), using tenfold serial dilutions of RLEP plasmid standards.

The LODs were re-confirmed in the matrix of 10 of the “must not detect RLEP” samples (7 swab samples, 2 slit-skin-smears and 1 biopsy sample; all RLEP qPCR and RLEP DRB LAMP negative), spiked with 1.000 copies of the plasmid standard. Therefore the “must not detect samples” amounted to one-half of the template volume and 2 × 10^3^ copies of the plasmid standard amounted to the other half to get the required dilution of plasmid standard in a semi-natural “sample”.

#### Sensitivity

The sensitivity rate was defined as the number of “must detect RLEP” clinical samples with a positive RLEP DRB LAMP divided by the number of all “must detect RLEP” clinical samples (n = 25).

#### Specificity

The specificity rate was defined as the number of “must not detect RLEP” samples with a negative RLEP DRB LAMP divided by the number of all “must not detect RLEP” samples (n = 40).

#### Inter-assay variation

Inter-assay variability was assessed by testing an RLEP plasmid standard (diluted 10^6^) on 3 subsequent days to calculate the mean, standard deviation (SD) and coefficient of variation (CV) of the inter-assay variability. Calculations were conducted in accordance with MIQE guidelines for qPCR in previously described modifications for other NAAT assays [1, 62, 64]. Variability was judged low if the coefficient of variation (CV) was ≤ 0.05, medium CV ≤ 0.2, high CV > 0.2.

#### Intra-assay variation

Seven samples with Std 10^6^ were analyzed in one run to calculate the mean, SD and CV of the intra-assay variability. Variability was judged low if the coefficient of variation (CV) was ≤ 0.1, medium CV ≤ 0.5, high CV > 0.5.

#### Semiquantitative results

The correlation between the bacillary load and the time to positivity (Tp) was assessed.

### Clinical validation

For clinical validation of both the in-house and the ready-to-use RLEP DRB LAMP formats, positivity and negativity rates, as well as positive and negative prediction values, were determined as follows:

#### Positivity rate

The positivity rate was defined as the number of RLEP qPCR positive samples with a positive RLEP DRB LAMP divided by the number of all RLEP qPCR positive samples (n = 90).

#### Negativity rate

The negativity rate was defined as the number of RLEP qPCR negative samples with a negative RLEP DRB LAMP divided by the number of all RLEP qPCR negative samples (n = 60).

#### Positive predictive value (PPV)

The PPV was defined as the number of all RLEP qPCR positive samples with a positive RLEP DRB LAMP divided by the number of all RLEP DRB LAMP positive samples.

#### Negative predictive value (NPV)

The NPV was defined as the number of all RLEP qPCR negative samples with a negative RLEP DRB LAMP divided by the number of all RLEP DRB LAMP negative samples.

### Statistical analysis

For comparison of the two LAMP formats, an estimation of the standard error of proportion (95-per cent confidence intervals [95%-CI]) was conducted with Microsoft Excel 2010 (Microsoft cooperation, Redmond, USA). Significant differences were defined as not overlapping of 95%-CI of proportions.

## Results

After optimizing the protocol, the RLEP DRB LAMP formats (in-house and ready-to-use) passed through storage testing and technical as well as clinical validation.

### LAMP—optimizing the protocol

Regarding the optimization of the “wet” RLEP LAMP protocol (the preliminary stage of both RLEP DRB LAMP formats), the first test revealed that the Isothermal Mastermix ISO-DR-004 proved to be more sensitive and rapid than the ISO-DR-001. For the second test, inner and outer primers at a ratio of 2:1 proved to be more sensitive than at a ratio of 1:1. For the third test, the temperature 65.0 °C proved to be the most sensitive. Based on these test results the optimized protocol as outlined in the “Methods” section was determined. Data are provided in Table 3.

**Table 3:** Results of the LAMP optimization

	Cq value [cycles]			Melting temperature [°C]		
	Mean	SD	CV	Mean	SD	CV
Test 1: master mix						
ISO-DR-001	21.78	0.13	0.006	91.90	0.00	0.000
ISO-DR-004	14.59	0.11	0.007	91.95	0.07	0.001
Test 2: primer ratio						
1:1	17.05	0.01	0.001	92.00	0.00	0.000
2:1	12.77	0.51	0.040	92.00	0.00	0.000
Test 3: annealing temperature (°C)						
68.6	18.64	3.42	0.183	92.20	0.28	0.003
68.1	19.21	–	–	92.00	–	–
67.0	19.15	2.42	0.126	92.15	0.21	0.002
65.0	15.97	0.74	0.047	92.00	0.00	0.000
62.6	19.24	0.89	0.046	91.95	0.07	0.001
60.6	21.76	0.98	0.045	92.00	0.14	0.002
59.3	24.26	0.59	0.024	91.95	0.07	0.001
58.6	28.41	1.81	0.064	91.85	0.07	0.001

### Storage testing

In-house storage performed well at 48 °C for up to 60 days, but standard samples were tested negative after storage at 48 °C for 120 days resulting in shelf life for 1 year at ambient temperature (22 °C) as calculated utilizing an accelerated ageing calculation [58]. All in-house RLEP DRB LAMP tubes stored at 4 °C performed well, resulting in the minimum shelf life of 1 year.

Ready-to-use RLEP DRB LAMP storage testing resulted in the shelf life of 18 months at room temperature according to the manufacturers’ instructions (15–30 °C).

### Technical validation for RLEP DRB LAMP (in-house and ready-to-use)

#### LOD

The lower limit of detection for both RLEP DRB LAMP formats was 1.000 RLEP copies, with an amplifiable copy number varying between 23 and 37 that constitute 43–27 genome equivalents (for Togolese patients 33 genome equivalents and for one patient with Pakistani origin 43 genome equivalents). The LOD was re-confirmed in the matrix of clinical samples.

#### Sensitivity

For the in-house RLEP DRB LAMP, 23/25 “must detect RLEP” clinical samples were tested positive, resulting in a sensitivity of 92% (95%-CI 81; 100). For the ready-to-use RLEP DRB LAMP, all 25 “must detect RLEP” clinical samples were tested positive, resulting in a sensitivity of 100%. Data are provided in Table 4.

**Table 4:** Testing of “must (not) detect RLEP” samples resulting in sensitivity and specificity of both RLEP DRB LAMP formats (in-house and ready-to-use)

	In-house RLEP DRB LAMP		Ready-to-use RLEP DRB LAMP	
	Positive	Negative	Positive	Negative
Samples				
Must detect RLEP	23	2	25	0
	Sensitivity 92%		Sensitivity 100%	
Must not detect RLEP	0	40	0	40
	Specificity 100%		Specificity 100%	

#### Specificity

For both RLEP DRB LAMP formats all of the 40 “must not detect” samples were tested negative, resulting in a specificity of 100% for the in-house as well as for the ready-to-use RLEP DRB LAMP. Data are provided in Table 4.

#### Inter-assay variation

For both RLEP DRB LAMP formats, the inter-assay variability was judged low concerning annealing temperature. Concerning time to positivity and fluorescence, the inter-assay variability was judged medium for the in-house RLEP DRB LAMP and low for ready-to-use RLEP DRB LAMP. Data are provided in Table 5.

**Table 5:** Data on inter-and intra-assay variability of the RLEP DRB LAMP formats (in-house and ready-to-use)

	Time to positivity (Tp) ( min)			Fluorescence (k)			Annealing temperature (°C)		
	Mean	SD	CV	Mean	SD	CV	Mean	SD	CV
In-house RLEP DRB LAMP									
Inter-assay	14:35	02:33	0.175	29.33	4.16	0.142	92.00	0.01	0.000
Intra-assay	16:08	00:56	0.059	21.86	0.69	0.032	91.79	0.09	0.001
Ready-to-use RLEP DRB LAMP									
Inter-assay	18:00	00:15	0.014	35.67	1.53	0.043	92.07	0.04	0.000
Intra-assay	18:04	00:24	0.022	35.71	1.80	0.050	92.04	0.07	0.001

*SD* standard deviation, *CV* coefficient of variation

#### Intra-assay variation

For both RLEP DRB LAMP formats, the intra-assay variability was judged low. Data are provided in Table 5.

### Clinical validation

Results of the testing of clinical samples are summarized in Table 6.

**Table 6:** Results of the clinical validation of both RLEP DRB LAMP formats (in-house and ready-to-use)

	RLEP qPCR positive samples			RLEP qPCR negative samples	
	High positive samples of MB leprosy patients	Medium positive samples of MB leprosy patients	Low positive samples of MB leprosy patients	Samples from endemic controls	Samples from healthy non-exposed controls
In-house RLEP DRB LAMP					
Positive result	30	30	23	0	0
Negative result	0	0	7	30	30
Proportion	100% pos	100% pos	77% pos	100% neg	100% neg
95%-CI	–	–	62; 92	–	–
Ready-to-use RLEP DRB LAMP					
Positive result	30	28	13	0	0
Negative result	0	2	17	30	30
Proportion	100% pos	93% pos	43% pos	100% neg	100% neg
95%-CI	–	84; 100	26; 61	–	–

#### Positivity rate—in-house RLEP DRB LAMP

For high positive samples, 100% (30/30) were tested positive. For medium positive samples, 100% (30/30) were tested positive. For low positive samples (i.e. samples below the LOD), 77% (23/30; 95%-CI 62; 92) were tested positive. The positivity rate for all samples tested was 92% (83/90, 95%-CI 87; 98). The positivity rate for all samples above the LOD was 100%. Analysis of the different sample types: out of seven false negative samples, one was a buccal swab sample (1/2, i.e. 50% of all positive buccal swab samples tested [95%-CI 0; 100]), five samples were nasal swab samples (5/74, i.e. 7% of all positive nasal swab samples tested [95%-CI 1; 12]) and one sample was a slit skin smear (1/13, i.e. 8% of all positive slit skin smears tested [95%-CI 0; 22]).

#### Positivity rate—ready-to-use RLEP DRB LAMP

For high positive samples, 100% (30/30) were tested positive. For medium positive samples, 93% (28/30, 95%-CI 84; 100) were tested positive. For low positive samples (i.e. samples below the LOD), 43% (13/30, 95%-CI 26; 61) were tested positive. The positivity rate for all samples tested was 79% (71/90, 95%-CI 70; 87). The positivity rate for all samples above the LOD was 97% (58/60, 95%-CI 92; 100). Analysis of the different sample types: of the 19 false negative samples, two were buccal swab samples (2/2, i.e. 100% of all positive buccal swab samples tested) and 17 were nasal swab samples (17/74, i.e. 23% of all positive nasal swab samples tested [95%-CI 13; 33]). Significant differences between both RLEP DRB LAMP formats were found for the positivity rates of all 90 positive samples tested as well as for the positivity rates for low positive samples.

#### Negativity rate

For both RLEP DRB LAMP formats, the negativity rate was 100% (30/30 samples from endemic controls and 30/30 samples from healthy non-exposed controls were negative).

#### Positive predictive value—in-house RLEP DRB LAMP

The positive predictive value was 100% (0/83 results were false positive).

#### Positive predictive value—ready-to-use RLEP DRB LAMP

The positive predictive value was 100% (0/71 results were false positive).

#### Negative predictive value—in-house RLEP DRB LAMP

The negative predictive value was 90% (7/67 results were false negative).

#### Negative predictive value—ready-to-use RLEP DRB LAMP

The negative predictive value was 76% (19/79 results were false negative).

#### Semi-quantitative results

**Fig. 3:**
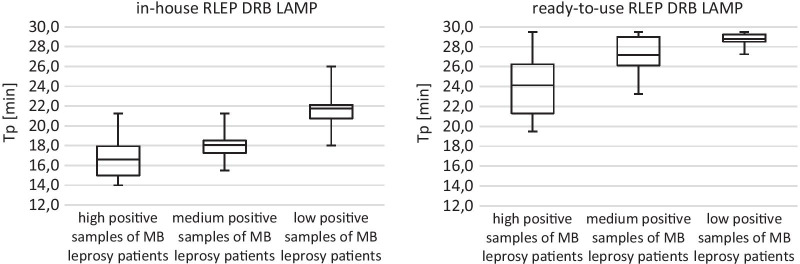
Comparison of the semi-quantitative results of RLEP DRB LAMP formats (in-house and ready-to-use). Tp = time to positivity; boxplot with the lowest and highest result shown as whiskers

Both RLEP DRB LAMP formats proved to be semi-quantitative, there is a negative correlation between the time to positivity (Tp) and the bacillary load. Data are provided in Table 7 and Fig. 3.

**Table 7:** Comparison of the semi-quantitative results of RLEP DRB LAMP formats (in-house and ready-to-use)

	High positive samples of MB leprosy patients			Medium positive samples of MB leprosy patients			Low positive samples of MB leprosy patients		
	Mean	SD	CV	Mean	SD	CV	Mean	SD	CV
In-house RLEP DRB LAMP									
Tp [mm:ss]	16:30	02:00	0.112	18:00	01:15	0.075	21:45	01:45	0.085
Ready-to-use RLEP DRB LAMP									
Tp [mm:ss]	24:15	03:00	0.113	27:00	02:00	0.068	28:45	00:45	0.026

*Tp* time to positivity, *SD* standard deviation, *CV* coefficient of variation

## Discussion

Nucleic acid-based amplification tests (NAAT) are widely used for the laboratory confirmation of TB and BUD in reference laboratories of endemic countries and constitute an integral part of disease control activities for both conditions [65, 66]. As demonstrated in recent publications, NAAT also have great potential for applications in leprosy control. NAAT enable laboratory-based early confirmation of leprosy cases, allow laboratory-based screening of HHC facilitating laboratory confirmation of difficult to diagnose lesions and identification of individuals with nasal carriage of *M. leprae* eligible for further monitoring, as well as assessing the treatment success [4, 5, 16, 17, 24, 25, 67, 68]. Due to the lack of standardization and commercial availability, the requirement of technical and laboratory expertise, and the difficulty to perform NAAT in most primary health-care settings, current leprosy control strategies have not yet entailed the application of NAAT and the WHO still recommends clinical examination, if available supported by microscopy, as the standard method for diagnosis of leprosy [1, 69]. The Global Leprosy Strategy 2021–2030, however, schedules implementation of laboratory diagnostics from 2026 onwards and the TAG explicitly recommends improving laboratory capacity including molecular diagnostics [27, 28].

Recent leprosy research prioritized early case detection, contact screening and chemoprophylaxis as the most promising tools to interrupt transmission and enhance the effectiveness of leprosy control.

Results from the most comprehensive study on combining contact tracing with prophylactic treatment “Leprosy Post-Exposure Prophylaxis” (LPEP) with single-dose rifampicin (SDR) conducted from 2015 through 2019 with the leprosy control programs of Brazil, India, Indonesia, Myanmar, Nepal, Sri Lanka, and Tanzania, showed that the approach of contact tracing followed by the provision of SDR is feasible as part of routine leprosy control activities. Contacts of clinically diagnosed leprosy index patients were traced and screened for signs of the disease. Those with signs of active leprosy were evaluated according to the routine leprosy control program procedures and, if the diagnosis was confirmed, were administered MDT. Contacts without any evidence of leprosy were assessed for their eligibility to receive SDR. The authors concluded that further research should focus on local epidemiological situations, i.e. the level of endemicity, the ratio of multibacillary to paucibacillary patients and health system characteristics. Following the WHO Global Leprosy Strategy, and the conclusions of other studies, the development of field-friendly diagnostic tests can help to identify early leprosy and those contacts with subclinical infection who would benefit most from SDR post-exposure prophylaxis or further measures, such as modified chemoprophylactic regimens or intensified monitoring [69–72].

In accordance with the current state of research, the priority to develop a POC test for leprosy is high [73]. A test suitable for the developing world should meet the WHO *ASSURED* criteria (*A*ffordable, *S*ensitive, *S*pecific, *U*ser-friendly, *R*apid and robust, *E*quipment-free and *D*elivered) [74, 75]. Classical NAAT such as (q)PCRs typically stand out in the fields of sensitivity and specificity but cannot meet the other criteria. LAMP comes up with several advantages: The technology is less expensive than other NAAT, has an equal sensitivity as classical PCR, a high specificity due to its multiple primers, is rapid (< 1 h turn-around time) and does not require sophisticated equipment [30, 50, 75, 76].

Although perfectly suitable for POC diagnostics, LAMP has a few disadvantages, especially the specificity is questioned: briefly, although multiple primers promote specificity, it may also increase the risk of primer-primer binding and therefore false-positive results. In this validation study, the authors used a modified melting curve analysis of each LAMP product to ensure the specificity of amplicons on the Genie^®^ III fluorometer (as provided by the manufacturer). Nevertheless vast technical and clinical validation studies are required prior to broad-range field-application [77, 78].

Like in other NAAT, unintended contamination through carryover of DNA from well-to-well is also of concern applying LAMP [78–81]. The extraction and run protocols should therefore be reduced to a minimum of pipetting steps, preferable in a closed, one-step system [36].

Nijiru et al. rated LAMP a potential ASSURED test, given four elements, i.e. template preparation protocols, a lyophilized kit, a reliable power source, and product detection technologies are available [76]. Template preparation was not in the focus of this study but must be subject to further, separate investigation. The other three elements were covered by the formats compared in this study. The formats were dry-reagent based (including master mix and primers) and therefore robust, affordable and worked on a closed amplification and detection unit with a stable power source sufficient for working one whole day in the field (the device only needs a standard power socket for charging). The fluorometric detection of LAMP products allowed the real-time visualization of the amplification curves and, based on the predetermined strand-inherent temperature profile, the application of a melting curve analysis presented a means of quality assurance.

Although coming close to the equipment-free principle put in place by the WHO 2012 [82], our assays were not completely equipment-free which may be considered a drawback. Lateral flow assays (LFAs) are typical examples of equipment-free POC tests, defining them as devices “operable in resource-limited environments, which include those with unreliable electricity, non-sterile conditions and a lack of trained personnel to perform the duties typically reserved for nurses and health workers” [82, 83]. With its stable power source and closed set-up the Genie^®^ III fluorometer meets part of the criteria, but although it is hand-held it represents technical equipment. According to the manufacturer the Genie^®^ III software may be programed based on our validation data to simply display the results as positive or negative, but the personnel working with it will still need at least a short training before fully understanding the LAMP assay. However, due to the option of melting curve analysis, the technical component provides the chance to rule out the above-mentioned disadvantage of primer-primer binding and false-positive results. In the authors’ view, the advantages outweigh the disadvantages.

Four LAMPs for detecting *M. leprae* were published up to now (Garg et al., Jiang et al., Joshi et al. and Joshi et al. m-LAMP), all 100% specific, with a sensitivity ranging from 83 to 100% (being more sensitive for MB patients and tissue samples and less sensitive for PB patients and slit skin smears) and an analytical sensitivity of 30 bacilli each for both LAMPs from Joshi et al. [51–54]. With a specificity of 100%, a sensitivity of 92% for minimal invasive samples (nasal swab samples and slit skin smears) and an analytical sensitivity of 27–43 bacilli (depending on the number of RLEP copies per strain) our results are well comparable with the other recently published assays. The three published LAMPs applying standard consumables and easy-to-use nontechnical equipment have two limiting factors: non-specific amplification with different primers can generate false-positive samples, which is a common drawback of LAMP and—as described by Jiang et al.—the fluorescence-based visual detection of results can be ambiguous, leading to the need of confirmation by gel electrophoresis, which would outweigh the benefits of the minimalist technique and can also generate false-positive samples due to contamination [51–53]. Joshi et al. solved these problems with their m-LAMP in the same way as we did, i.e. with a melting curve analysis to rule out false-positive samples and a closed and precise detection system [54]. However, in contrast to our DRB assays, the reagents for all *M. leprae* detecting LAMP formats recently described need to be transported at lower temperatures (mostly at − 20 °C), which hampers the use for POC.

A POC test must be stable at ambient temperature for as long as possible. DRB LAMP assays are mostly stabilized through drying [84–86] or lyophilization [87–89]. Several LAMPs have been reported to be stable at 4 °C for 2–5 months [37, 87] and at ambient temperature (specified in the literature as 15–30 °C) for 3 weeks to 7 months [37, 84, 87, 88, 90]. To the best of our knowledge, with (more than) 1 year both LAMP assays revealed the longest shelf-life at 4 °C and 22 °C. It is, therefore, possible to e.g. produce the test in Europe, ship it to endemic countries at ambient temperature via normal postal service for storage and application at POC without loss of sensitivity for test results. To the best of our knowledge, this is unique to all previously described *M. leprae* LAMP assays.

Our two RLEP DRB LAMP formats differ only by the mode of lyophilization (in-house lyophilization vs commercial lyophilization by Amplex directly in the wells of the LAMP strips for the ready-to-use kit). But despite the substantial common features both formats show differences: The in-house RLEP DRB LAMP is significantly superior in detecting samples with a low bacillary load of *M. leprae* as positive, but requires in-house lyophilization, which is time-consuming and costly (concerning the devices needed). The ready-to-use RLEP DRB LAMP on the other hand is with one less pipetting step easier to prepare and more protected against contamination, and—once CE-certified—may be purchased in large quantities directly from the producer.

Application of the RLEP DRB LAMP assays at POC of course rises the question of the relevance of positive results for classification and clinical management of patients. The WHO classification introduced in 1988 initially employed the lesion count and the bacteriological index (BI) for distinction of PB and MB forms [91]. With the abolishment of bacteriological examination in 1998, misclassification occurred and studies describe that up to 51% of cases clinically diagnosed as PB forms were in fact MB cases—with the consequence of undertreatment and the risk of treatment failure [92, 93]. The limit of detection of microscopy is indicated as 10^4^ bacilli per g tissue which corresponds to approximately 100 bacilli in a 6 mm punch biopsy [94, 95]. The limit of detection of our RLEP DRB LAMP assay has been determined approximately 30 bacilli per sample, the sensitivity of the assay is therefore three times higher as the sensitivity of microscopy. In terms of clinical application the assays can provide a bacteriological confirmation of PB cases, especially those with scanty bacilli (BI 1) with a higher sensitivity than microscopy could achieve.

Furthermore, according to the previous approach of applying lesion numbers and bacteriological examination to classification, clinically diagnosed PB cases with LAMP positivity could be correctly re-classified as MB cases and treated accordingly. We assume that a number of BT cases with scanty bacilli which are in fact correctly classified as PB cases by counting of lesions will be detected as well—but we believe that “overtreatment” in sporadic BT cases is acceptable and less of an issue of concern as “undertreatment” of missed MB cases. However, to put these assumptions on a sound scientific basis, detailed studies on the correlation of clinical classification by lesion count, BI, RLEP DRB LAMP positivity in different sample types (nasal swabs, SSS, biopsies) and histopathological classification are required which use RLEP qPCR as reference standard to determine threshold values for the bacillary load associated with different histological forms of the disease.

An evaluation trial in leprosy endemic regions may be required, especially for answering the question if HHC with subclinical infection are also detected by the assays. Thereby, the ready-to-use RLEP DRB LAMP may serve as large scale screening test for HHC as well as clinically suspected leprosy cases. Due to its augmented positivity rates in medium- and low-positive samples, the in-house RLEP DRB LAMP, which is lyophilized manually and therefore only in low quantities, should be only used as confirmatory test, if the first test reveals a negative result despite strong clinical evidence of leprosy. In this step-wise-approach RLEP qPCR remains the third NAAT, conducted if both LAMP results turn out to be negative. Notwithstanding the capacity of NAAT, clinical diagnosis should remain the key component to administer adequate therapy if WHO criteria are met.

The ready-to-use RLEP DRB LAMP format presented here constitutes an ASSURED test applicable at the primary health care level and can facilitate early case detection of leprosy index cases without the need for a third level reference laboratory [33]. Financing provided, a field-based evaluation trial to determine the clinical performance at peripheral health care level in Togo using the previously established infrastructure [60, 96], will provide ideal conditions for evaluation of the ready-to-use RLEP DRB LAMP prototype for leprosy regarding project logistics and availability of trained personnel.

In collaboration with FIND, a DRB IS*2404* LAMP kit for Buruli ulcer using identical laboratory techniques as the ready-to-use RLEP DRB LAMP is currently on the way to commercial production. While the specificity of the IS*2404* LAMP equals that of ready-to-use RLEP DRB LAMP for clinical samples, the analytical sensitivity was higher. For *M. ulcerans* the LOD was 50 copies of the target sequence (i.e. 0.25 M*. ulcerans* genome equivalents) [55] compared to 1.000 copies for RLEP DRB LAMP (i.e. 27–43 *M. leprae* genome equivalents according to the geographic origin and occurring RLEP variants). In terms of genome equivalents, this fact is explained by the vastly higher copy number of IS*2404* per *M. ulcerans* genome (i.e. n = 209 [66]) as compared to RLEP per *M. leprae* genome (n = 23–37) [13–17]. Furthermore, the modified *M. ulcerans* LAMP [55] employed an additional set of loop-primers as first described by Ablordey et al. enhancing the process of amplification and thus the sensitivity [31]. However, we did not succeed in designing loop-primers encompassing nucleotide regions of all RLEP variants.

The new LAMP assays for laboratory diagnosis of leprosy and BUD expand the existing spectrum of NAAT for neglected tropical diseases from 16 to 18 pathogens and will be of great help for the managing of the respective diseases. The implementation of nearly identical LAMP solutions for two related mycobacterial diseases is a good example of the advantages of the integrated management of skin diseases, which result in the synergistic use of laboratory space, equipment and personnel in settings where both diseases are endemic, as it is already commonplace in Togo.

## Conclusions

The priority to develop a POC test for leprosy in line with the *ASSURED* criteria is high. Based on our own experience with the development of a dry-reagent-based (DRB) LAMP for decentralized diagnosis of Buruli ulcer, our group designed and validated the first RLEP DRB LAMP assay for the diagnosis of leprosy at point-of-care. The DRB LAMP assay is *affordable*, 100% *sensitive*, 92–100% *specific* and has an LOD of 43–27 genome equivalents. It runs on the Genie^®^ III (OptiGene), a *user-friendly* and *rapid* (run time 45 min) portable fluorometer. The dry-reagent-based assay is *robust* and stable for 12 months at ambient temperature. It is coming close to the *equipment-free* principle put in place by the WHO 2012 and—once CE-certified—can be *delivered* to point-of-care by simply purchasing the device (OptiGene) with lyophilized LAMP strips and the accompanying buffer (Amplex). The ready-to-use RLEP DRB LAMP assay was finally validated for field-based evaluation trials aiming for routine POC testing of leprosy suspected cases.

## Supplementary Information

**Additional file 1. **In-house “wet” RLEP LAMP run protocol.

**Additional file 2. **In-house lyophilization protocol.

**Additional file 3. **In-house RLEP DRB LAMP run protocol.

**Additional file 4. **Ready-to-use RLEP DRB LAMP run protocol.

## Data Availability

The datasets used and/or analyzed during the current study are available from the corresponding author on reasonable request.

## Abbreviations

AFB: Acid fast bacilli
BI: Bacteriological index
BL: Bacillary load
BLAST: Basic local alignment search tool
*Bst*: *Bacillus stearothermophilus*
BUD: Buruli ulcer disease
CE: “Conformité Européenne”, administrative marking
CI: Confidence interval
CLS: Cell lysis solution
Cp: Copies
CV: Coefficient of variation
DITM: Division of Infectious Diseases and Tropical Medicine
DRB: Dry-reagent-based
FIND: Foundation for Innovative Diagnostics
FNA: Fine needle aspirate
HAT: Human African trypanosomiasis
HHC: Household contact
IC: Informed consent
INH: Institut National d’Hygiène
KCCR: Kumasi Centre for Collaborative Research in Tropical Medicine
KNUST: Kwame Nkrumah University of Science and Technology
LAMP: Loop-mediated isothermal amplification
LFA: Lateral flow assay
LMU: Ludwigs-Maximilians-University of Munich
LOD: Lower limit of detection
MB: Multibacillary
m-LAMP: Multiplex LAMP
*M. leprae*: *Mycobacterium leprae*
NAAT: Nucleic acid-based amplification tests
PB: Paucibacillary
POC: Point-of-care
SD: Standard deviation
SDR: Single-dose rifampicin
SQ: Starting quantity
TAG: Technical and Advisory Group
TB: Tuberculosis
Tp: Time to positivity
