# Supplementary material for: RLEP LAMP for the laboratory confirmation of leprosy: towards a point-of-care test
Source: BMC Infect Dis. 2021 Nov 25;21:1186. doi: 10.1186/s12879-021-06882-2 (PMC8620619; doi:10.1186/s12879-021-06882-2)
Supplement: Supplementary file 1 — Additional file 1. In-house “wet” RLEP LAMP run protocol. [file 12879_2021_6882_MOESM1_ESM.pdf]

RLEP LAMP for the laboratory confirmation of leprosy: towards a point-of-care test

BMC Infectious Diseases

Malkin Saar, Marcus Beissner, Fatih Gültekin, Issaka Maman, Karl-Heinz Herbinger and Gisela Bretzel

Additional file 1: in-house "wet" RLEP LAMP run protocol

**Assay:**	**RLEP LAMP**
**Analysis:**	**Detection of *Mycobacterium leprae* DNA (RLEP)**
**Type of NAAT:**	**Loop-mediated isothermal amplification**
**Type of Primers:**	**Wet**

**Outer Primer:** (5 µM)	
**RLEP F3**	5'CGCACCTGATGTTATCCCTT'3
**RLEP B3**	5'GGTTTGGGTGGTGTTGTGG'3

**Inner Primer:** (20µM)	
**RLEP FIP**	5'ATGCCTGCTTGCTGGCTGAG CACCATTTCTGCCGCTGG'3
**RLEP BIP**	5'CAGTGCATCGATGATCCGGCC GTGTGGGTGGTTGATCTGC'3

Amount of samples: **6 samples, 1 positive control, 1 negative control = 8 (plus 20% additive due to pipetting lost)**

**Reagent**	**Single reaction [µl]**	**Master mix [µl]**	**Concentration of stock solution**	**Final concentration**
Isothermal Master mix ISO-DR-004 [Optigene; Horsham, UK]	15.00	**150.00**	-	1x
Primer F3	1.00	**10.00**	5 µM	0.2 µM
Primer B3	1.00	**10.00**	5 µM	0.2 µM
Primer FIP	2.00	**20.00**	20 µM	1.6 µM
Primer BIP	2.00	**20.00**	20 µM	1.6 µM
H_2O	2.00	**20.00**	-	-
Total vol. reaction mix [µl]	23.00	**230.00**		
Template	2.00			
Final volume	25.00			

Amplification/ Annealing

1	65°C	30 min.

Melting curve

2	80°C – 98.5°C

Detection: **FAM channel**
Device: **Genie III**

**Date:**		**Description:**				
**Well**	**Sample**	**Material**	**Result**	**Time to positivity [min:ss]**	**Fluoroscence [k]**	**Annealing temperature [°C]**
1						
2						
3						
4						
5						
6						
7						
8						

Results interpretation: Result positive if amplification ≤25 min AND fluorescence ≥ 5 [k]
Result negative if amplification >25min OR fluorescence < 5 [k]
