# Supplementary material for: RLEP LAMP for the laboratory confirmation of leprosy: towards a point-of-care test
Source: BMC Infect Dis. 2021 Nov 25;21:1186. doi: 10.1186/s12879-021-06882-2 (PMC8620619; doi:10.1186/s12879-021-06882-2)
Supplement: Supplementary file 2 — Additional file 2. In-house lyophilization protocol. [file 12879_2021_6882_MOESM2_ESM.pdf]

RLEP LAMP for the laboratory confirmation of leprosy: towards a point-of-care test

BMC Infectious Diseases

Malkin Saar, Marcus Beissner, Fatih Gültekin, Issaka Maman, Karl-Heinz Herbinger and Gisela Bretzel

Additional file 2: in-house lyophilization protocol

Protocol:
One vial Isothermal Master mix ISO-DR-004 [Optigene; Horsham, United Kingdom] equals 16 tubes with lyophilized reagents for in-house DRB LAMP (please refer to additional file 3).
It is of great importance to work quickly with the master mix as soon as it is dissolved in water and until it is frozen. Otherwise it will degrade fast.

Day 1: preparation

- Prepare the lyoadapter from the lyophilization device by freezing to -20°C
- Label 16 lyotubes (0.5ml screw cap micro tubes [SARSTEDT, Nümbrecht, Germany])
- Prepare primer mix:
  870µl (290µl FIP+290µl BIP+ 145µl F3+ 145µl B3), mix
- pipette 54 µl primer mix in each of the 16 tubes
- Prepare master mix: pipette 1500µl H_2O in one vial Isothermal Master Mix ISO-DR-004 and let it dissolve completely, shake gently
  Carry on as soon as it is dissolved!
- Quickly pipette 90µl master mix in each of the 16 tubes
- Zentrifuge the tubes shortly and put them into the prepared Lyoadapter (-20°C)
- Store Lyoadapter including tubes overnight at -80°C

Day 2: lyophilization

- Start the lyophilization device (e.g. Alpha 1-2 LDplus [Christ, Osterode, Germany])
- Start “warm up”
- Fetch the lyoadapter including tubes from -80°C and open all the tubes
- Put the lyoadapter including tubes in the device and close the device
- Start drying program
- Let the tubes dry at 0.31 mbar for 20 hours

Day 3: end phase

- After ~20h: start the subsequent drying phase
- Let it run for ½-1 hour until it reaches 0.011 mbar or does not go further down
- Open device carefully and slowly
- Close all tubes
- Store lyophilized reaction mix dry and dark at 4°C or room temperature
  Stable for 12 months at RT and >12 months at 4°C

Lyotest

- Always test the newly lyophilized batch by running one LAMP test (please refer to additional file 3).
- Instead of samples a RLEP standard curve as described in the main paper should be used.
