# Supplementary material for: RLEP LAMP for the laboratory confirmation of leprosy: towards a point-of-care test
Source: BMC Infect Dis. 2021 Nov 25;21:1186. doi: 10.1186/s12879-021-06882-2 (PMC8620619; doi:10.1186/s12879-021-06882-2)
Supplement: Supplementary file 3 — Additional file 3. In-house RLEP DRB LAMP run protocol. [file 12879_2021_6882_MOESM3_ESM.pdf]

# RLEP LAMP for the laboratory confirmation of leprosy: towards a point-of-care test

BMC Infectious Diseases

Malkin Saar, Marcus Beissner, Fatih Gültekin, Issaka Maman, Karl-Heinz Herbinger and Gisela Bretzel

## Additional file 3: in-house RLEP DRB LAMP run protocol

| | |
|---|---|
| **Assay:** | **RLEP LAMP** |
| **Analysis:** | **Detection of *Mycobacterium leprae* DNA (RLEP)** |
| **Type of NAAT:** | **Loop-mediated isothermal amplification** |
| **Type of Primers:** | **Dry reagent based (lyophilization in-house, please refer to additional file 2)** |

| **Outer Primer**: (5 µM) | |
|---|---|
| **RLEP F3** | 5'CGCACCTGATGTTATCCCTT'3 |
| **RLEP B3** | 5'GGTTTGGGTGGTGTTGTGG'3 |

| **Inner Primer**: (20µM) | |
|---|---|
| **RLEP FIP** | 5'ATGCCTGCTTGCTGGCTGAG CACCATTTCTGCCGCTGG'3 |
| **RLEP BIP** | 5'CAGTGCATCGATGATCCGGCC GTGTGGGTGGTTGATCTGC'3 |

**Amount of samples:** **6 samples, 1 positive control, 1 negative control = 8 (+10% additive due to pipetting lost)**

| **Reagent** | **Single reaction [µl]** | **Master mix [µl]** |
|---|---|---|
| Dryed master mix | - | **1 tube** |
| Buffer ISO-DR-004 | 15.00 | **135.00** |
| $H_2O$ | 8.00 | **72.00** |
| Total vol. reaction mix [µl] | 23.00 | **207.00** |
| Template | 2.00 | |
| Final volume | 25.00 | |

**Amplification/ Annealing**

| | | |
|---|---|---|
| 1 | 65°C | 30 min. |

**Melting curve**

| | |
|---|---|
| 2 | 80°C – 98.5°C |

**Detection:** **FAM channel**

**Device:** **Genie III**

| **Date:** | **Description:** | | | | | |
|---|---|---|---|---|---|---|
| **Well** | **Sample** | **Material** | **Result** | **Time to positivity [min:ss]** | **Fluoroscence [k]** | **Annealing temperature [°C]** |
| 1 | | | | | | |
| 2 | | | | | | |
| 3 | | | | | | |
| 4 | | | | | | |
| 5 | | | | | | |
| 6 | | | | | | |
| 7 | | | | | | |
| 8 | | | | | | |

**Results interpretation: Result positive if amplification ≤25 min AND fluorescence ≥ 5 [k]**
**Result negative if amplification >25min OR fluorescence < 5 [k]**
