# Supplementary material for: RLEP LAMP for the laboratory confirmation of leprosy: towards a point-of-care test
Source: BMC Infect Dis. 2021 Nov 25;21:1186. doi: 10.1186/s12879-021-06882-2 (PMC8620619; doi:10.1186/s12879-021-06882-2)
Supplement: Supplementary file 4 — Additional file 4. Ready-to-use RLEP DRB LAMP run protocol. [file 12879_2021_6882_MOESM4_ESM.pdf]

RLEP LAMP for the laboratory confirmation of leprosy: towards a point-of-care test

BMC Infectious Diseases

Malkin Saar, Marcus Beissner, Fatih Gültekin, Issaka Maman, Karl-Heinz Herbinger and Gisela Bretzel

Additional file 4: ready-to-use RLEP DRB LAMP run protocol

**Assay:**	**RLEP LAMP**
**Analysis:**	**Detection of *Mycobacterium leprae* DNA (RLEP)**
**Type of NAAT:**	**Loop-mediated isothermal amplification**
**Type of Primers:**	**Dry reagent based (lyophilization by Amplexdiagnostics GmbH, Germany)**

**Outer Primer**: (5 µM)	
**RLEP F3**	5'CGCACCTGATGTTATCCCTT'3
**RLEP B3**	5'GGTTTGGGTGGTGTTGTGG'3

**Inner Primer**: (20µM)	
**RLEP FIP**	5'ATGCCTGCTTGCTGGCTGAG CACCATTTCTGCCGCTGG'3
**RLEP BIP**	5'CAGTGCATCGATGATCCGGCC GTGTGGGTGGTTGATCTGC'3

	**Amount of samples:**	**6 samples, 1 positive control, 1 negative control = 8**
**Reagent**	**Single reaction [µl]**	No reaction mix, pipette directly into the wells of the delivered strips.
dryed master mix strips	Already prepared in the well	
resuspension solution RS	12.50	
H_2O	7.50	
Total vol. in well [µl]	20.00	
Template	5.00	
Final volume	25.00	

Amplification/ Annealing

1	65°C	30 min.

Melting curve

2	80°C – 98.5°C

**Detection:**	**FAM channel**
**Device:**	**Genie III**

**Date:**		**Description:**				
**Well**	**Sample**	**Material**	**Result**	**Time to positivity [min:ss]**	**Fluoroscence [k]**	**Annealing temperature [°C]**
1						
2						
3						
4						
5						
6						
7						
8						

Results interpretation:

Result positive if annealing temperature 91.5 ±1°C AND Tp >10 min AND fluorescence ≥ 5 [k]

If fluorescence >2: Use the Genie Explorer: Result positive if annealing temperature 91.5 ±1°C AND starting fluorescence ≥ 20 [k] AND Tp >10 min (threshold 0.002 ratio)

Result negative if otherwise
